# Oxyresveratrol Induces Autophagy via the ER Stress Signaling Pathway, and Oxyresveratrol-Induced Autophagy Stimulates MUC2 Synthesis in Human Goblet Cells

**DOI:** 10.3390/antiox9030214

**Published:** 2020-03-05

**Authors:** Jiah Yeom, Seongho Ma, Young-Hee Lim

**Affiliations:** 1 Department of Integrated Biomedical and Life Sciences, Graduate School, Korea University, Seoul 02841, Korea; intro56@naver.com (J.Y.); aktjdgh8@naver.com (S.M.); 2 Department of Public Health Sciences (Brain Korea 21 PLUS program), Graduate School, Korea University, Seoul 02841, Korea; 3 Department of Laboratory Medicine, Korea University Guro Hospital, Seoul 08308, Korea

**Keywords:** autophagy, oxyresveratrol, ER stress, mucin, goblet cell

## Abstract

Background: Autophagy is a cell protection system invoked to eliminate the damaged organelles and misfolded proteins that induce various stresses, including endoplasmic reticulum (ER) stress. Autophagy can control mucin secretion in goblet cells. Oxyresveratrol (OXY), an antioxidant, stimulates expression of MUC2. Thus, we investigated the effect of OXY on autophagy and found that OXY-induced autophagy stimulates MUC2 expression in human intestinal goblet cells. Methods: Autophagy-related genes and proteins were examined by quantitative real-time PCR (qPCR) and Western blotting, respectively. Autophagy was assessed by immunocytochemistry (ICC). To analyze the protein expression profiles of OXY-treated LS 174T goblet cells, two-dimensional electrophoresis (2DE) and peptide mass fingerprinting (PMF) were performed. MUC2 expression in cells was evaluated by ICC. Results: OXY significantly increased the expression levels of genes related to autophagy induction, and activated phagosome elongation resulted in the formation of autophagosomes. OXY also activated the ER stress signaling pathway and promoted MUC2 synthesis, which was inhibited by treatment with an autophagy inhibitor. Conclusion: OXY induces autophagy via the ER stress signaling pathway, and OXY-induced autophagy increases MUC2 production in intestinal goblet cells.

## 1. Introduction

Autophagy is a self-digesting process that is characterized by the removal of damaged organelles, misfolded proteins, and old or nonfunctional proteins [1,2]. This system is a type of survival mechanism that is triggered in response to diverse stresses, such as starvation and hypoxia, to maintain intracellular homeostasis. The primary role of autophagy is protection against cell death, and the process has a role in stress management, immunity, and preventing various disorders such as cancer [3], diabetes, and obesity [4]. Autophagy deficiency promotes tumorigenesis [5,6] and causes neurodegenerative diseases [7] and chronic inflammation [8].

Autophagy is divided into three types according to the mechanisms by which cargo is transported to the lysosomes or vacuoles. Macroautophagy is a mechanism that delivers cargo to the lysosome through autophagosomes that are composed of double membrane-bound vesicles. In microautophagy, cargo is directly transported to the lysosome itself. Chaperone-mediated autophagy (CMA) is a mechanism that delivers chaperone-selected proteins to the lysosome membrane for degradation [1]. Among them, macroautophagy is the major form of autophagy and is characterized by the formation of an autophagosome that is composed of a microtubule-associated protein 1 light chain 3 (LC3B)-phosphatidylethanolamine (PE) complex and an autophagy-related gene (ATG) complex [9]. The formation of autophagosomes is initiated by unc-51-like kinase (ULK) and class III phosphatidylinositol 3-kinase (PI3K), leading to the isolation of a membrane called a phagophore. Autophagosomes subsequently become elongated and closed by the LC3B-II-PE and Atg5-Atg12-Atg16L1 complexes. As LC3B-I is converted to LC3B-II during this step, this conversion of LC3B-I to LC3B-II is the most widely used marker for monitoring autophagy activity [2].

MUC2 is a major factor of the intestinal mucus layer that is a critical protection system in the intestine, and mucus is secreted by goblet cells in the intestinal epithelium. Destruction of the mucus layer induces intestinal inflammation, which might cause inflammatory bowel disease (IBD) classified as ulcerative colitis (UC) and Crohn’s disease (CD) [10,11]. Thus, maintenance of the integrity of the mucus layer is important for protecting against intestinal inflammation.

A stilbenoid, oxyresveratrol (OXY), has multiple beneficial effects, such as anti-inflammatory [12], antibacterial activity [13], and intestinal tight junction integrity enhancement [14]. OXY shows strong antioxidant activity [15]. In our previous study, we found that *Ramulus mori*, derived from *Morus alba* L. (mulberry), contain a high content of OXY and that an ethanolic extract significantly attenuated colitis by suppressing inflammation as well as increasing mucin production [16]. Additionally, we found that OXY stimulates mucin production by increasing the synthesis of NAD^+^ in human goblet cells [17]. NAD^+^ protects cells by upregulating autophagy [18], and autophagy promotes mucin secretion [19]. Therefore, we hypothesized that OXY might enhance mucin production by increasing autophagic activity. In this study, we investigated the effect of OXY on autophagy-stimulated mucin production and elucidated its mechanism in the mucin producing human goblet cells.

## 2. Materials and Methods

### 2.1. Materials

Roswell Park Memorial Institute (RPMI) medium, fetal bovine serum (FBS), and penicillin/streptomycin for cell culture were purchased from HyClone (Logan, UT, USA). MTT (3-[4–dimethylthiazol-2-yl]-2,5-diphenyltetrazolium bromide) was purchased from Amresco (Solon, OH, USA). Dimethyl sulfoxide (DMSO), 3-methyladenine (3-MA), sodium phenylbutyrate (4-PBA), and oxyresveratrol (OXY) were purchased from Sigma-Aldrich (St. Louis, MO, USA). U0126 (a MEK1/2 inhibitor), SP600125 (a JNK1/JNK2 inhibitor), and SB203580 (a p38 MAPK inhibitor) were obtained from Selleckchem (Houston, TX, USA). CYTO-ID^®^ Autophagy Detection Kit (ENZ-51031) was obtained from Enzo Life Science (Farmingdale, IL, USA).

### 2.2. Cell Culture

The human LS 174T goblet cell line was obtained from the Korea Cell Line Bank (KCLB, Seoul, Korea). The cells were cultured in RPMI-1640 medium supplemented with 10% FBS, 100 units/mL penicillin, and 100 µg/mL streptomycin and incubated in an atmosphere of 5% CO_2_-95% air at 37 °C. The cells were seeded in appropriate plates when confluence reached approximately 70–80%.

### 2.3. MTT Measurement of OXY Cytotoxicity

Cells were seeded in 96-well plates at a density of 2.5 × 10^5^ cells/mL and incubated overnight at 37 °C. OXY was dissolved in DMSO; the final concentration of DMSO in the cell culture medium was maintained below 0.5%. The cells were treated with OXY at 2.5, 5, 10, and 20 µg/mL for 24 h, the medium was aspirated, and MTT diluted 1:40 in cell medium was added. After incubation for 1 h at 37 °C, unreacted MTT was removed, and the formazan crystals formed were dissolved in DMSO for 1 h at room temperature. Absorbance at 540 nm was measured using a SpectraMax 340PC384 plate reader (Molecular Devices, Sunnyvale, CA, USA), and cell viability (%) was calculated as a percentage relative to the untreated negative group.

### 2.4. Quantitative Real-Time Polymerase Chain Reaction (qPCR)

LS 174T cells were seeded in 6-well plates at a density of 2.5 × 10^5^ cells/mL and treated with 10 µg/mL OXY for 24 h. For inhibition assays, the inhibitor was added 1 h before treatment with OXY. Total RNA was extracted using TRIzol reagent (Bioneer, Daejon, Korea) according to the manufacturer’s instructions. RNA was quantified using a Nanodrop ND-1000 spectrophotometer (Thermo Scientific, Wilmington, DE, USA). RNA was converted to cDNA using a RevertAid First Strand cDNA Synthesis kit (Thermo Fisher Scientific, Waltham, MA, USA). qPCR was performed with the Kapa SYBR Fast qPCR kit (Kapa Biosystems, Woburn, MA, USA) using StepOnePlus™ Real-time PCR System (Applied Biosystems, Foster City, CA, USA). Glyceraldehyde-3-phosphate dehydrogenase (*GAPDH*) was applied as the internal control gene, and the primer sequences used are shown in Table 1. The reaction was as follows: 95 °C for 10 min followed by 40 cycles of 95 °C for 15 s, 60 °C for 15 s, and 72 °C for 20 s. Relative gene expression was quantified based on equal amounts of RNA (1 μg). The normalized expression change is expressed as 2^-ΔΔCt^ (the *GAPDH* control was set to 1) [20].

### 2.5. Western Blot Analysis

LS 174T cells were cultured with 10 µg/mL OXY in 60-mm dishes for 18 h at 37 °C. As mentioned above, inhibitor was added 1 h before treatment with OXY for inhibition assays. The cells were harvested using 0.25% trypsin-EDTA and lysed for protein extraction using the Pro-Prep protein extraction solution (Intron, Seoul, Korea). Total protein concentrations were determined by the Bradford assay. Equal amounts (10 µg for Beclin-1, Atg7, and IRE1α; 20 µg for Atg5, LC3B, ERK, and phosphorylated ERK) of proteins for each sample were separated by 10% sodium dodecyl sulfate-polyacrylamide gel electrophoresis (SDS-PAGE), except for LC3B, which was separated by 12% SDS-PAGE. The separated proteins were transferred to a polyvinylidene difluoride (PVDF) membrane (Millipore, Bedford, MA, USA) using a Trans-Blot semi-dry transfer cell (Bio-Rad, Hercules, CA, USA). The membrane was blocked with 5% nonfat skim milk in phosphate-buffered saline (PBS) with 0.05% Tween 20 (PBS-T) at room temperature for 1 h, washed with PBS-T or Tris-buffered saline with 0.05% Tween 20 (TBS-T), and incubated overnight at 4 °C with primary antibodies. Antibodies against the endogenous control β-actin (1:5000 dilution, MA5-15739, Thermo Scientific, Wilmington, DE, USA), IRE1α (1:1000 dilution, sc-390960, Santa Cruz Biotechnology, Dallas, TX, USA), Beclin-1 (1:1000 dilution, ab62557, Abcam, Cambridge, UK), Atg7 (1:100,000 dilution, ab52472, Abcam), Atg5 (1:1000 dilution, ab108327, Abcam), LC3B (1:1000 dilution, ab51520, Abcam, Cambridge, UK), ERK (1:1000 dilution, sc-514302, Santa Cruz Biotechnology, Dallas, TX, USA), and phosphorylated ERK (1:1000 dilution, sc-81492, Santa Cruz Biotechnology, Dallas, TX, USA) were used. The membrane was washed with PBS-T or TBS-T and incubated with the secondary antibody for 1 h at room temperature. A goat anti-mouse IgG (H+L) horseradish peroxidase-conjugated antibody (1:10,000 dilution, NCI1430KR, Thermo Scientific, Wilmington, DE, USA) was used as the secondary antibody for anti-β-actin, anti-IRE1α, anti-ERK, and anti-phosphorylated ERK, and A goat anti-rabbit IgG (H+L) horseradish peroxidase-conjugated antibody (1:5000 dilution, NCI1460KR, Thermo Scientific, Wilmington, DE, USA) was used as the secondary antibody for anti-Beclin-1, anti-Atg7, anti-Atg5, and anti-LC3B. After incubation with the secondary antibody, the membrane was washed with PBS-T or TBS-T and detected with the SuperSignal West Femto Maximum Sensitivity Substrate kit (Thermo Fisher Scientific, Wilmington, DE, USA). Blot images were obtained and analyzed using a FluorChem E imaging system (Proteinsimple, San Jose, CA, USA).

### 2.6. Two-Dimensional Electrophoresis (2DE) and Peptide Mass Fingerprinting (PMF)

Cultured cell pellets were washed twice with ice-cold PBS, and protein samples were prepared by using 2DE lysis solution composed of 7 M urea, 2 M thiourea containing 4% (w/v) 3-[(3-cholamidopropy) dimethyammonio]-1-propanesulfonate (CHAPS), 1% (w/v) dithiothreitol (DTT), 2% (v/v) Pharmalyte, and 1 mM benzamidine. The proteins were extracted by vortexing followed by centrifugation at 12,000 rpm for 1 h at 25 °C. The supernatant was used for 2DE. For isoelectric focusing (IEF), immobilized pH gradient (IPG) strips were equilibrated for 12-16 h with a reswelling solution composed of 7 M urea, 2 M thiourea containing 2% CHAPS, 1% DTT, and 1% Pharmalyte. Each sample (800 μg) was examined, and IEF was performed at 20 °C using a Multiphor II electrophoresis unit and an EPS 3500 XL power supply (Amersham Biosciences, Piscataway, NJ, USA) following the manufacturer’s instructions. Prior to SDS-PAGE, the strips were incubated in equilibration buffer (pH 6.8, 50 mM Tris-HCl, 6 M urea, 2% SDS, and 30% glycerol) for 10 min, first with 1% DTT and then with 2.5% iodoacetamide. The equilibrated strips were arrayed onto SDS-PAGE gels (20 × 24 cm, 10–16%), and SDS-PAGE was performed using a Hoefer DALT 2D system (Amersham Biosciences). The 2D gels were fixed, rehydrated, and stained with Sypro ruby staining solution (Invitrogen, Carlsbad, CA, USA). After staining, the gels were visualized as described by Anderson et al. [21], and quantitative analysis of digitized images was carried out using the PDQuset software (version 7.0, BioRad). Protein spots that significantly increased over two-fold compared with the negative control were selected.

For protein identification by PMF, protein spots were excised, digested with trypsin (Promega, Madison, WI, USA), mixed with α-cyano-4-hydroxycinnamic acid in 50% acetonitrile/0.1% trifluoroacetic acid (TFA), and subjected to matrix-assisted laser desorption/ionization time-of-flight mass spectrometry (MALDI-TOF) analysis (Microflex LRF 20, Bruker Daltonics, Billerica, MA, USA), as described by Fernandez et al. [22]. The search program MASCOT, developed by Matrixscience (http://www.matrixscience.com/), was used for protein identification by PMF.

### 2.7. Autophagy Detection

LS 174T cells were seeded at a density of 1.25 × 10^5^ cells/mL on a coverslip with 0.1% gelatin in 24-well plates and incubated with various concentrations of OXY at 37 °C for 18 h. The cells were stained with CYTO-ID^®^ Green Detection Reagent (Enzo Life Sciences, Farmingdale, IL, USA) for 30 min at 37 °C and then washed with assay buffer according to the manufacturer’s instructions. Nuclei were counterstained with Hoechst 33342 (Enzo Life Science, Farmingdale, IL, USA). Autophagic vacuole accumulation and flux were both detected by fluorescence microscopy using a Nikon C1 plus confocal laser scanning microscope (Nikon, Tokyo, Japan).

### 2.8. Immunocytochemistry (ICC)

For the measurement of MUC2 protein expression, cells were fixed in 4% paraformaldehyde and permeabilized with 0.01% Triton X-100 in PBS. The cells were blocked with 10% normal donkey serum (GTX73205, Genetex, Irvine, CA, USA) and incubated at 4 °C overnight with the primary antibody against MUC2 (1:1000 dilution; GTX100664, Genetex, Irvine, CA, USA). Goat anti-rabbit IgG, DyLight 488 (35553, Thermo Scientific) diluted 1:1000 in 2% normal donkey serum was used as the secondary antibody. 4′,6-Diamidino-2-phenylindole (DAPI) (1:10,000 dilution, Sigma, St. Louis, MO, USA) was employed to counterstain the nuclei for 5 min at room temperature. Coverslips were mounted using VECTASHIELD^®^ (Vector Laboratories, Burlingame, CA, USA). Images were obtained, and the fluorescence intensity was quantified using a Nikon C1 plus confocal laser scanning microscope.

### 2.9. Statistical Analysis

All statistical analyses were performed using the Statistical Package for the Social Sciences (SPSS, Chicago, IL, USA) version 24.0. Data are expressed as the mean ± standard deviation (SD) of three independent experiments. The statistical significance of differences between samples was determined with Student’s *t*-test. Statistical differences among groups were determined using one-way analysis of variance (ANOVA) followed by post hoc Tukey’s HSD (honestly significant difference) test. A *p* value of < 0.05 was considered statistically significant.

## 3. Results

### 3.1. Cytotoxicity of OXY in LS 174T Goblet Cells

**Figure 1:**
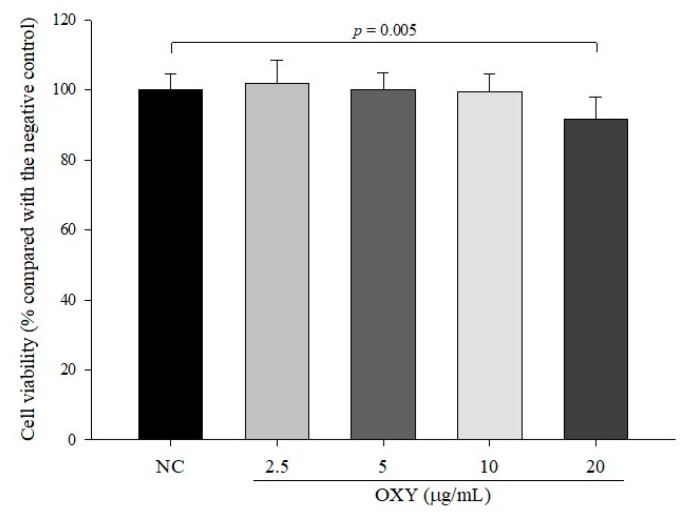
Cytotoxicity of oxyresveratrol (OXY) in LS 174T goblet cells. Cell viability was measured by MTT assays after 24 h of treatment with OXY. Data are indicated the mean ± SD of three independent experiments performed in triplicate.

The cytotoxic effect of OXY on LS 174T goblet cells was evaluated after treatment with OXY for 24 h using the MTT assay. The relative viabilities of cells treated with 2.5, 5, 10, and 20 µg/mL OXY were 101.7 ± 6.7%, 100.1 ± 4.7%, 99.4 ± 5.1%, and 91.6 ± 6.1%, respectively, compared with the negative control (Figure 1). As the viability of the cells treated with 20 µg/mL OXY was significantly reduced, we used 2.5, 5, and 10 µg/mL OXY for ensuing experiments in this study.

### 3.2. Effect of OXY on Autophagy Development at Specific Times

**Figure 2:**
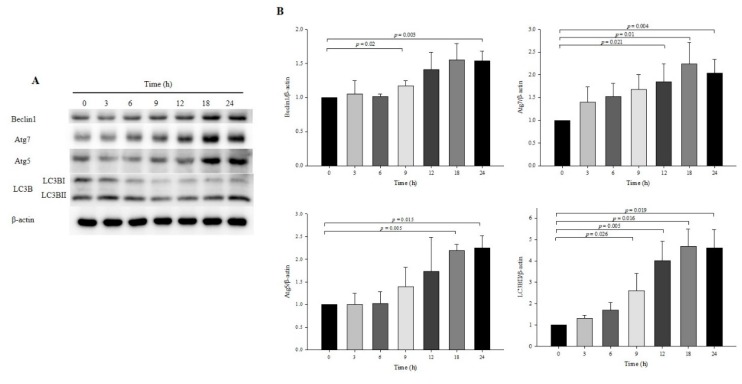
Effect of OXY on autophagy development at various time points. Cells were treated with 10 μg/mL OXY, and expression levels of autophagy-associated proteins (Beclin-1, Atg7, Atg5, and LC3B-II) were determined by Western blotting (**A**) and quantified (**B**). From each lysate, equal amounts of protein were loaded on a separate gel, blotted for actin and this signal was used for determining the ratio protein of interest/actin as displayed in the figure. The cropped blots are representative of three independent experiments. The full-length blots are shown in Supplementary Figure S1. Each value indicates the mean ± SD of three independent experiments performed.

Time is an important factor in autophagy induction; thus, observation of autophagy at a specific time point is susceptible to inaccurate interpretations [23,24]. To determine the appropriate time to induce autophagy by treatment with OXY, the expression levels of autophagy-related proteins were measured by Western blotting at various time points. OXY (10 μg/mL) significantly increased the protein expression of Atg7, Atg5, and LC3B at 18 and 24 h but Beclin-1 significantly increased only at 18 h (Figure 2). Therefore, subsequent assays were performed at 18 h or 24 h.

### 3.3. OXY Increases Expression of Autophagy-Related Factors

**Figure 3:**
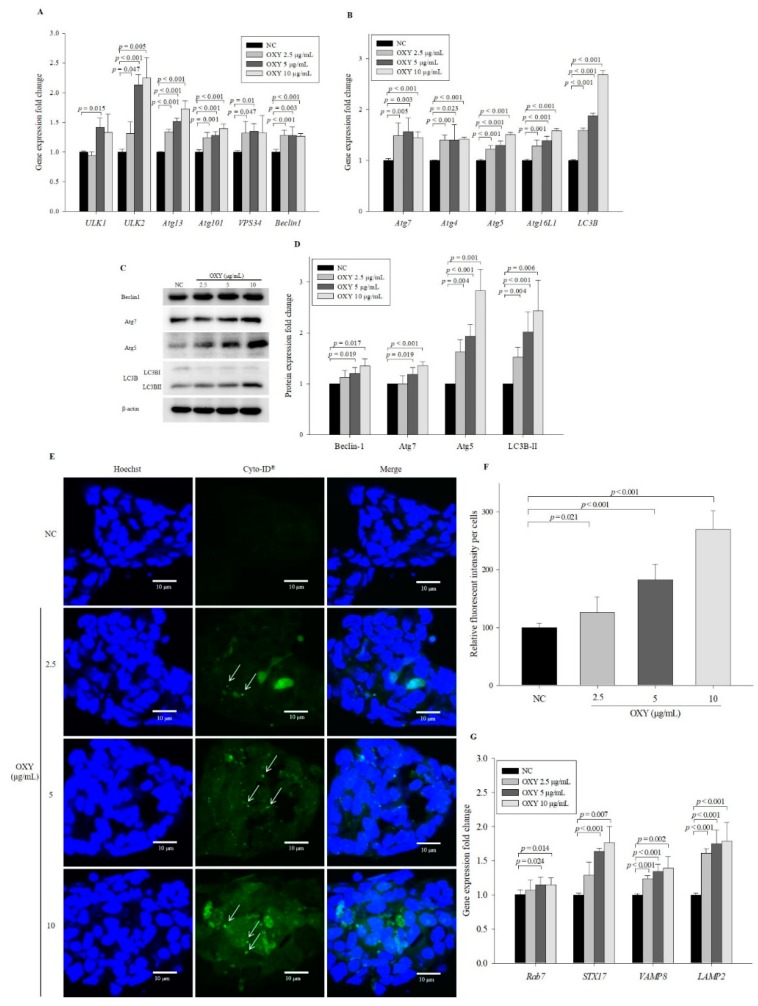
Effect of OXY on the expression levels of autophagy-related genes and proteins. Expression levels of genes related to phagophore formation were quantified by qPCR (**A**). Expression levels of autophagosome elongation-related genes were quantified by qPCR (**B**). Expression levels of autophagosome formation-related proteins were measured by Western blotting (**C**) and quantified (**D**). From each lysate, equal amounts of protein were loaded on a separate gel, blotted for actin and this signal was used for determining the ratio of the protein of interest/actin as displayed in the figure. The cropped blots are representative of three independent experiments. The full-length blots are shown in Supplementary Figure S2. The effect of OXY on autophagic vesicle formation was measured by staining with a fluorescence dye using a CYTO-ID autophagy detection kit; white arrows indicate autophagosomes (magnification 600×) (**E**), which were quantified (**F**). Expression levels of autolysosome formation-related genes were quantified by qPCR (**G**). Each value indicates the mean ± SD of three independent experiments performed in triplicate.

Phagophore formation is initiated by activation of the ULK complex consisting of ULK1, ULK2, Atg13, and Atg101 as well as Beclin-1, which plays a critical role in the initial step in phagophore formation [25]. To investigate the effect of OXY on the early stage of phagophore formation, the expression levels of phagophore formation-related genes were measured by qPCR after 10 μg/mL OXY treatment for 18 h. Levels of *ULK2*, *Atg13*, *Atg101*, and *Beclin-1* in 2.5, 5, and 10 µg/mL OXY-treated cells increased significantly in a dose-dependent manner compared with the negative control (Figure 3A). Levels of *ULK1* and *VPS34* were also significantly increased in 5 µg/mL OXY-treated cells compared with negative control cells. These results suggest that OXY induces autophagy.

Autophagosome elongation results in the formation of a double-membrane vesicle, which is mainly driven by the Atg5-Atg12-Atg16L1 complex and LC3B conjugation system [1,2]. To investigate the effect of OXY on autophagosome elongation, the expression levels of genes that induce double-membrane vesicle formation were assessed. Compared with the negative control, expression levels of *Atg7*, *Atg4*, *Atg5*, *Atg16L1*, and *LC3B* were significantly elevated by OXY at 2.5, 5, and 10 µg/mL in a dose-dependent manner (Figure 3B), as were the protein expression levels of Beclin-1, Atg7, Atg5, and LC3B-II, which is converted from LC3B-I (Figure 3C,D). To further investigate the effect of OXY on autophagic vesicle formation, cells were treated with a green fluorescence dye that stains autophagic vacuoles in live cells. Significantly increased fluorescent signals were observed in cells treated with OXY in a dose-dependent manner, particularly with regard to increased punctate structure formation (arrows in Figure 3E), compared with the negative control (Figure 3E,F). The formation of green puncta indicates that LC3 was recruited to form autophagosomes [23]. The number of puncta increased with 10 μg/mL OXY treatment, whereas no puncta were observed in the negative control. These results suggest that OXY effectively enhances the formation of autophagosomes followed by autophagy induction.

The ultimate objective of autophagy is degradation of internal material after fusion with the lysosome [26]. To investigate the effect of OXY on autolysosome formation, the expression levels of genes related to autolysosome formation were measured after OXY treatment for 24 h. Levels of *Rab7*, *STX17*, *VAMP8*, and *LAMP2* significantly increased in cells treated with 2.5, 5, and 10 µg/mL OXY in a dose-dependent manner compared with the negative control (Figure 3G). These results show that OXY induces autophagosome–lysosome fusion by stimulating the expression of genes related to autolysosome formation.

### 3.4. 3-Methyladenine (3-MA) Decreases the Effect of OXY on Autophagy

**Figure 4:**
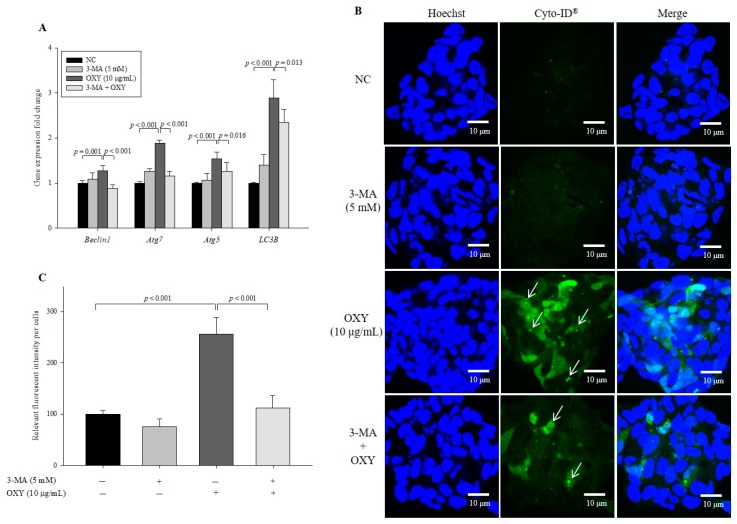
Effect of an autophagy inhibitor on OXY-induced autophagy. Cells were treated with OXY (10 μg/mL) with or without the autophagy inhibitor 3-MA, and expression levels of autophagy-related genes were determined by qPCR (**A**). Autophagic vesicle formation was detected by the CYTO-ID autophagy green dye, as observed by fluorescence microscopy; white arrows indicate autophagosomes (magnification 600×) (**B**), which were quantified (**C**). Each value indicates the mean ± SD of three independent experiments performed in triplicate.

To confirm the effect of OXY on autophagy, we used 3-MA, which inhibits autophagy by blocking the early stage of autophagosome formation, and found that expression levels of phagosome-related genes decreased significantly in cells cotreated with OXY and 3-MA compared with those treated with OXY alone (Figure 4A). In addition, the level of autophagic vesicle formation in cells cotreated with OXY and 3-MA decreased by 56.3% compared with the cells treated with OXY alone (Figure 4B,C). Interestingly, the significant inhibitory effect of 3-MA on autophagy was not observed in cells treated with 3-MA alone compared with the negative control, which may be because autophagy is less active under basal conditions. Therefore, the results suggest that OXY is an inducer of autophagosome formation.

### 3.5. OXY Stimulates the Mitogen-Activated Protein Kinase (MAPK) Signaling Pathway

**Figure 5:**
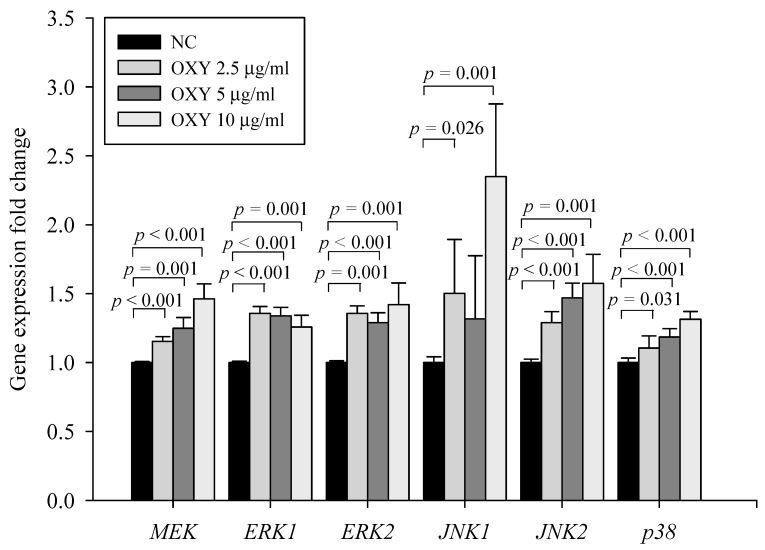
Effect of OXY on expression of MAPK signaling pathway-related genes. mRNA expression levels of *MEK*, *ERK1*, *ERK2*, *JNK1*, *JNK2*, and *p38* were determined by qPCR. Each value indicates the mean ± SD of three independent experiments performed in triplicate.

To examine the mechanism by which OXY induces autophagy, we investigated whether OXY affects the MAPK pathway, a potential autophagy induction pathway. The levels of *MEK*, *ERK1*, *ERK2*, *JNK1*, *JNK2*, and *p38* expression increased significantly in 2.5, 5, and 10 µg/mL OXY-treated cells (Figure 5). Accordingly, the results demonstrate that OXY stimulates the MAPK signaling pathway.

### 3.6. Effect of MAPK kinase Inhibitors on Expression Levels of Autophagy-Related Genes and Proteins

**Figure 6:**
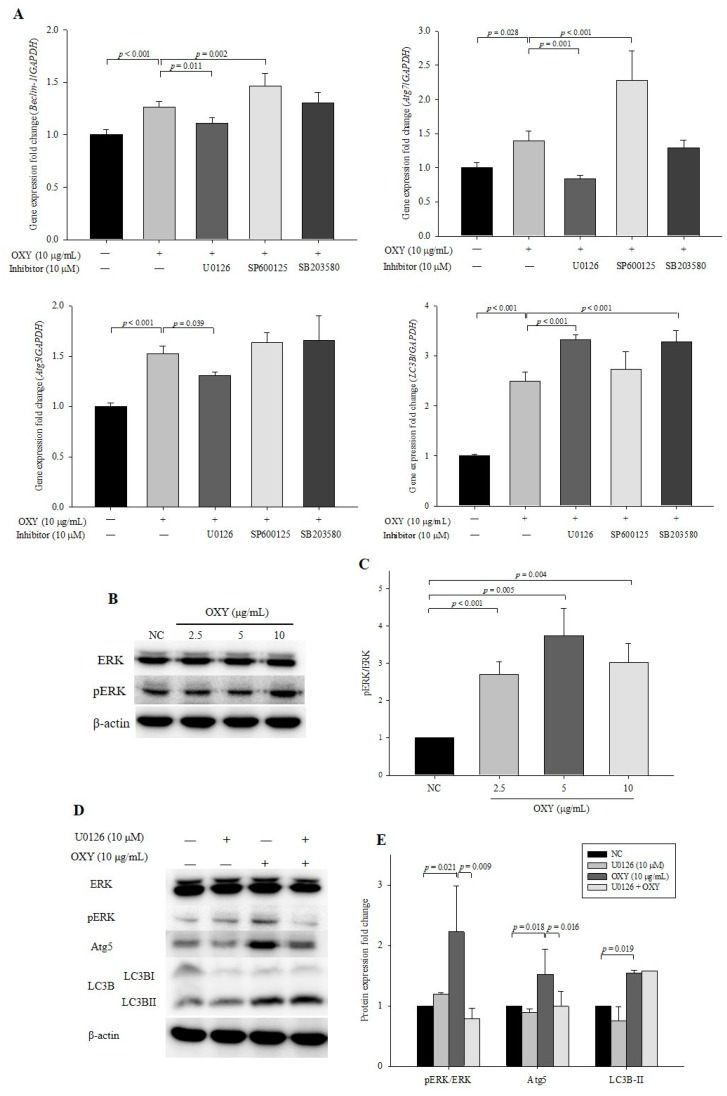
Effect of MAPK kinase inhibitors on the expression of autophagy-related genes and proteins. Cells were treated with OXY (10 μg/mL) with or without MEK inhibitor (U0126), JNK inhibitor (SP600125), and p38 inhibitor (SB203580). Expression levels of *Beclin-1*, *Atg7*, *Atg5*, and *LC3B* were determined by qPCR (**A**). Expression levels of phosphorylated ERK protein were measured by Western blotting (**B**) and quantified (**C**). Expression levels of phosphorylated ERK, Atg5, and LC3B proteins were measured by Western blotting (**D**) and quantified (**E**). From each lysate, equal amounts of protein were loaded on a separate gel, blotted for actin and this signal was used for determining the ratio of the protein of interest/actin as displayed in the figure. The cropped blots are representative of three independent experiments. The full-length blots are shown in Supplementary Figures S3 and S4. Each value indicates the mean ± SD of three independent experiments performed in triplicate.

To confirm that the OXY-stimulated MAPK signaling pathway is involved in autophagy, inhibitors of MEK/ERK (U0126), JNK (SP600125), and p38 (SB203580) were applied. In cells cotreated with OXY and U0126, expression levels of *Beclin-1*, *Atg5*, and *Atg7* decreased significantly compared with OXY-only-treated cells, whereas the level of *LC3B* increased in cells cotreated with OXY and U0126. In contrast, cotreatment with OXY and SP600125 or SB203580 did not decrease gene expression (Figure 6A). Interestingly cotreatment with OXY and SP600125 significantly increased expression levels of *Beclin-1* and *Atg7*, which might indicate that OXY and an JNK inhibitor show somehow a costimulatory effect on autophagosome formation. The lack of an inhibitory effect on *Beclin-1*, *Atg5*, and *Atg7* after exposure to SB203580 indicates that OXY-enhanced p38 signaling did not have autophagic effects. Thus, we only used U0126 in subsequent analyses to investigate whether OXY increases autophagic effects through the MEK/ERK signaling pathway.

Based on our gene expression data, we measured the level of ERK protein expression and found significant increases in phosphorylated ERK in 2.5, 5, and 10 µg/mL OXY-treated cells compared with the negative control (Figure 6B,C). We further investigated the expression levels of LC3B-I and LC3B-II, which revealed that U0126 significantly decreased the levels of phosphorylated ERK and Atg5 proteins (Figure 6D,E). Conversely, U0126 had no significant inhibitory effect on LC3B-II protein expression. The results suggest that Atg5, which forms a complex with other Atg proteins and participates in autophagosome membrane elongation, is stimulated through MEK/ERK signaling induced by OXY treatment but that LC3B, which accounts for the major complex of double-membraned autophagosomes and is most widely used as an autophagy marker, is not associated.

### 3.7. OXY Stimulates the ER Stress Signaling Pathway

**Figure 7:**
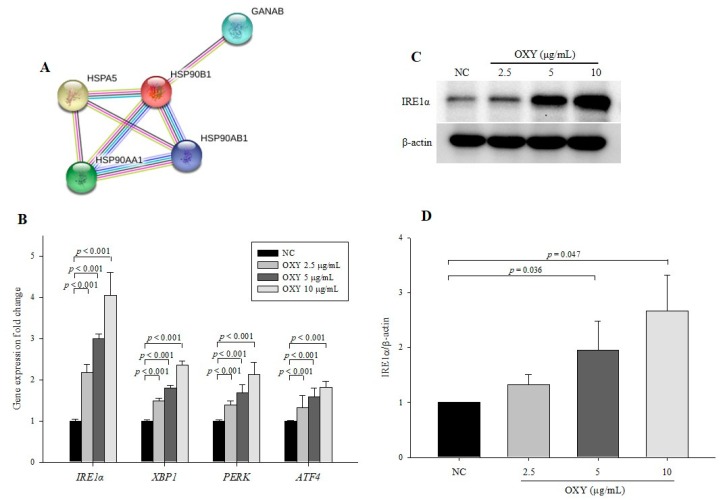
Effect of OXY on the expression levels of endoplasmic reticulum (ER) stress signaling pathway-related genes and proteins. Protein–protein interaction images were displayed using STRING (**A**). Expression levels of *IRE1α*, *XBP1*, *PERK*, and *ATF4* were determined by qPCR (**B**). The expression level of the IRE1α protein was measured by Western blotting (**C**) and quantified (**D**). From each lysate, equal amounts of protein were loaded on a separate gel, blotted for actin, and this signal was used for determining the ratio of the protein of interest/actin as displayed in the figure. The cropped blots are representative of three independent experiments. The full-length blots are shown in Supplementary Figure S5. Each value indicates the mean ± SD of three independent experiments performed.

To further elucidate the mechanism by which OXY stimulates autophagy, we performed 2DE by selecting protein spots showing significant expression variation that deviated by over two-fold compared with the negative control. In total, 13 proteins were identified by MALDI-TOF-MS (Table 2). The expression level of AP-1 complex subunit beta-1 (AP1B1) was markedly increased compared with other proteins. AP1B1 is found in the trans-Golgi network and mediates recruitment of clathrin, which interacts with Atg16L1 and is involved in the formation of early autophagosome precursors [27]. Among the 13 proteins, 5 (PHB, GANAB, HSPA5, HSP90B1, and HSP90AA1) are associated with protein processing in the endoplasmic reticulum (ER) according to Kyoto Encyclopedia of Genes and Genomes (KEGG) pathways and a network of the 5 protein interactions based on STRING, a meta-database program that generates a network of protein interactions from high-throughput experimental data, is shown in Figure 7A. The ER stress response is a protective process that is triggered by the accumulation of misfolded proteins in the ER. As ER stress is known to induce autophagy, we explored the effect of OXY on the expression of ER stress-related genes and proteins. OXY significantly increased *IRE1α*, *XBP1*, *PERK*, and *ATF4* mRNA expression in a dose-dependent manner compared with the negative control (Figure 7B). Furthermore, protein expression of IRE1α, which is the most conserved factor of the ER stress signaling branch, was significantly increased in OXY-treated cells in a dose-dependent manner compared with the negative control (Figure 7C,D).

### 3.8. An ER Stress Inhibitor Decreases Expression Levels of Autophagy-Related Genes and Proteins

**Figure 8:**
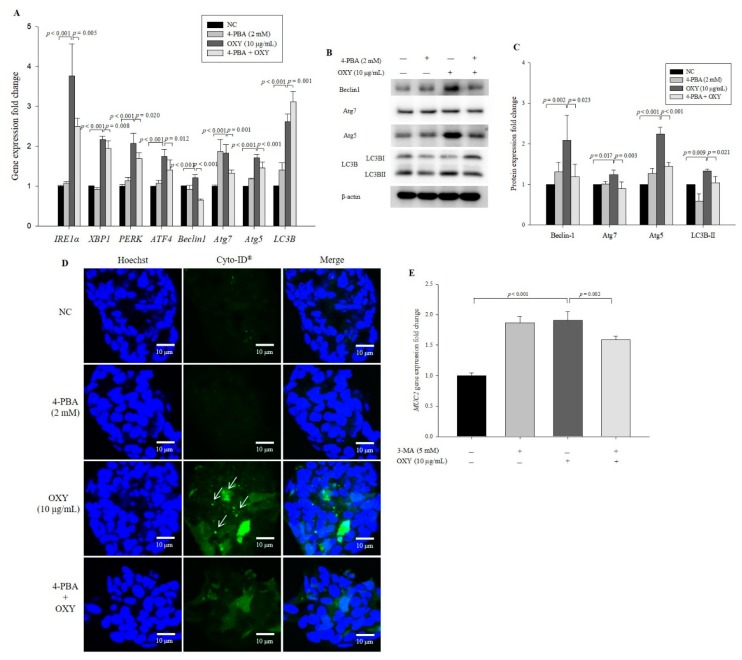
Effect of an ER stress inhibitor on expression of autophagy-related genes and proteins. Cells were treated with OXY (10 μg/mL) with or without the ER stress inhibitor 4-PBA. Expression levels of *IRE1α*, *XBP1*, *PERK*, *ATF4*, *Beclin-1*, *Atg5*, *Atg7*, and *LC3B* were determined by qPCR (**A**). Expression levels of Beclin-1, Atg7, Atg5, and LC3B proteins were measured by Western blotting (**B**) and quantified (**C**). From each lysate, equal amounts of protein were loaded on a separate gel, blotted for actin and this signal was used for determining the ratio of the protein of interest/actin as displayed in the figure. The cropped blots are representative of three independent experiments. The full-length blots are shown in Supplementary Figure S6. Autophagic vesicle formation was detected using the CYTO-ID autophagy green dye and observed by fluorescence microscopy; white arrows indicate autophagosomes (magnification 600×) (**D**), which were quantified (**E**). Each value indicates the mean ± SD of three independent experiments performed in triplicate.

To investigate whether the effect of OXY on autophagy involves the ER stress signaling pathway, 4-PBA that is a well-known ER stress inhibitor was used. Although *ATF4* levels did not decrease significantly, levels of *Beclin-1*, *Atg7*, *Atg5*, *IRE1α*, *PERK*, *ATE4*, and *XBP1* were significantly decreased in cells cotreated with OXY and 4-PBA compared with cells treated only with OXY; in contrast, 4-PBA did not inhibit expression of *LC3B* (Figure 8A). However, unlike U0126, 4-PBA significantly suppressed the conversion of LC3B-I to LC3B-II (Figure 8B,C). Cotreatment with OXY and 4-PBA also significantly decreased the levels of Beclin-1, Atg7, and Atg5 expression compared with the treatment with OXY alone. Moreover, the degree of autophagic vesicle formation decreased significantly by 42.3% in cells cotreated with OXY and 4-PBA compared with OXY-only-treated cells (Figure 8D,E). These results suggest that the effect of OXY on autophagy might be associated with ER stress signaling.

### 3.9. OXY-Induced Autophagy Affects Expression of MUC2

**Figure 9:**
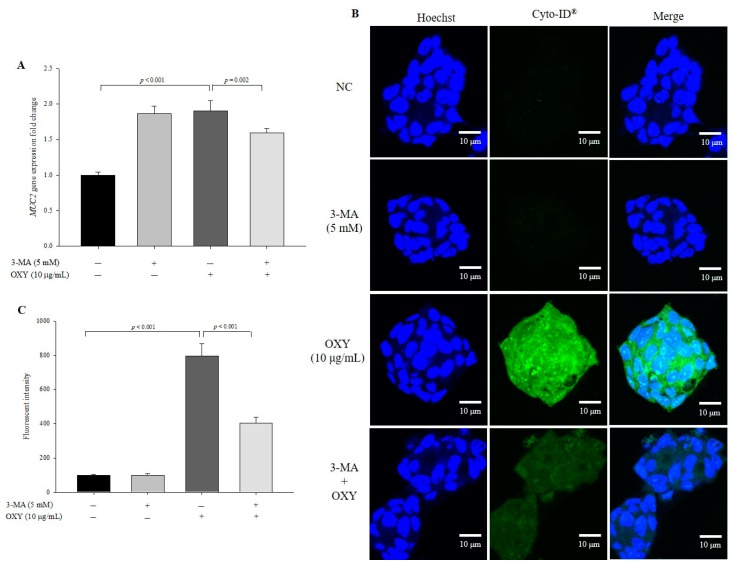
Effect of OXY on mRNA and protein expression of MUC2. Cells were treated with OXY (10 μg/mL) with or without the autophagy inhibitor 3-MA. The expression level of *MUC2* was measured by qPCR (**A**). The expression level of the MUC2 protein was determined by ICC (**B**) and quantified (**C**). Each value indicates the mean ± SD of three independent experiments performed in triplicate.

Goblet cells are colon epithelial cells that specialize in mucin production, and OXY stimulates MUC2 expression [17]. To investigate whether OXY-induced autophagy stimulates MUC2 production, the expression level of MUC2 was measured in cells treated with OXY with or without the autophagy inhibitor 3-MA. OXY significantly increased the level of *MUC2* expression, which was significantly decreased in cells cotreated with OXY and 3-MA (Figure 9A). Although expression level of *MUC2* increased in 3-MA only-treated cells, the increase was not statistically significant compared with the negative control. Similarly, the level of MUC2 protein expression decreased significantly by 49.3% in cells cotreated with OXY and 3-MA compared with OXY only-treated cells (Figure 9B,C). These results show that cotreatment of OXY with 3-MA significantly decreased OXY-dependent MUC2 expression. Unlike gene expression, the expression level of the MUC2 protein did not rise in cells treated with 3-MA alone compared with the negative control. This low correlation between mRNA and protein expression patterns may be because 3-MA inhibits PI3K protein expression. Therefore, OXY stimulates MUC2 expression, which might be associated with autophagy induction by OXY.

## 4. Discussion

OXY shows higher antioxidant activity than does resveratrol, a well-known antioxidant [15]. Antioxidants such as resveratrol, quercetin, and vitamin C protect cells and tissues against free radicals that cause damage to cells, proteins, and DNA. Thus, antioxidant supplementation can significantly improve the immune system against homeostatic disturbances [28,29,30]. The immune system is a complicated and exquisite network that is able to maintain homeostasis under normal physiological conditions [31], and antioxidants play a role in immune homeostasis. One of the mechanisms for maintaining homeostasis is the autophagy system, which induces the degradation of malfunctioning cellular molecules via autophagosomes and lysosomes [32]. Resveratrol induces autophagy by directly inhibiting the mTOR signaling pathway in HeLa cells [33], and OXY activates autophagy via inhibition of PI3K/AKT/mTOR and activation of p38 MAPK signaling pathways in neuroblastoma cells [34]. Although mTOR induces antioxidant activity, both reports did not show any direct evidences on autophagy induction by antioxidant properties of resveratrol and OXY. In general, as ROS increase, ER stress is induced. OXY treatment did not induce ROS production in neuroblastoma cells, which means that OXY-induced autophagy might be independent of antioxidant property of OXY. Therefore, further studies are needed to verify whether antioxidant property of OXY induces autophagy or OXY-induced autophagy leads to reduction of ROS production. In this study, we found that OXY induces autophagy in intestinal goblet cells through the ER stress signaling pathway, which might enhance mucin production.

In the autophagy process, a flat membrane (phagophore) is formed in response to various stimuli by two protein complexes; one complex is the ULK complex consisting of Atg13, Atg101, ULK1/2, and FIP200, and the other complex is the PI3K complex comprising Atg14L, Beclin1, Vps34, Vps15, and Ambra1 [35]. Under normal conditions, the ULK kinase complex is activated by the inhibition of mTOR, and formation of the isolation membrane using plasma membranes, the ER, and the Golgi apparatus is initiated [36,37]. The PI3K complex then continues to form a phagophore. Beclin-1, which contains a BH-3 domain, a central coiled-coil domain (CCD), and an evolutionarily conserved domain (ECD), plays a central role in regulating phagophore formation. Under normal conditions, the Bcl-2 (B-cell lymphoma 2) protein inhibits autophagy by interacting with the BH-3 domain in Beclin-1. However, when this interaction is disrupted, Beclin-1 alternatively binds with Vps34 and Vps15 through the CCD and ECD [38]. In the present study, OXY treatment increased the expression of genes related to phagophore formation. In particular, OXY significantly increased the Beclin-1 protein expression. Additionally, OXY increased mRNA expression of genes associated with autophagosomes. Autophagosome formation results from elongation of the phagophore, and this organelle is formed via conjugation of Atg5-Atg12-Atg16L1 and LC3B, a major autophagosomal marker. First, Atg12 conjugates with Atg5 with the aid of Atg7 and Atg10; next, Atg12-Atg5 forms the Atg5-Atg12-Atg16L1 complex located on the outer surface of the autophagosomal membrane. Second, phosphatidylethanolamine (PE)-conjugated LC3B-II, which is converted from LC3B-I, participates in the development of the inner and outer surfaces of the autophagosome, and autolysosome formation is completed by fusion with lysosomes. In this study, we demonstrate that OXY enhances mRNA expression of genes associated with each step of autophagy development. In particular, OXY significantly increased the expression of LC3B-II, which is widely used for the evaluation of autophagy activity in a dose-dependent and time-dependent manner. Overall, OXY activates autophagy from the initial step of autophagy induction, which was confirmed by using the autophagy inhibitor 3-MA.

Diverse signaling pathways participate in the induction of autophagy in mammalian cells [39], among which the MAPK pathway is notable. Three MAPK families, ERK, JNK, and p38 kinases, have been well characterized as being involved in many biological processes by regulating actions such as apoptosis, proliferation, and signaling cascades in mammalian cells [40]. In our previous study, OXY improved tight junction integrity through the MAPK signaling pathway in a colonic epithelial cell line [14]. Resveratrol and OXY upregulate autophagy via the MAPK signaling pathway. Resveratrol induces apoptosis and autophagy by inhibiting Akt/mTOR and activating the p38-MAPK pathway [41]. It is reported that resveratrol exerts neuroprotective effects by modulating mitochondrial dynamics and upregulating autophagic flux via the MEK/ERK pathway [42]. OXY also changes the activity of p38 MAPK and PI3K/AKT/mTOR, which induces apoptosis cell death independent of autophagy [34]. In our study, OXY stimulated the expression levels of genes related to MAPK kinases (*ERK, JNK,* and *p38*); however, when cells were cotreated with the respective inhibitors, except for the ERK inhibitor, gene expression was not inhibited. OXY promoted phosphorylation of the ERK1/2 protein, and the expression level of ERK decreased by cotreatment with OXY and the MAPK/ERK kinase inhibitor U0126. Interestingly, U0126 addition attenuated the effect of OXY on Atg5 expression at both transcriptional and translational levels; nonetheless, inhibition of MEK/ERK signaling did not decrease the LC3B level after OXY exposure. The results suggest that OXY-dependent Beclin-1, Atg7, and Atg5 stimulation may partially occur via the MEK/ERK signaling pathway but that OXY-induced autophagy might ultimately not be regulated by MAPK kinase. The results suggest that activated Atg5 functions in non-autophagic roles, as demonstrated previously [43,44]. Further studies are needed to explain why and how the MEK/ERK pathway is involved in only Atg5 expression but not in that of LC3B.

Our 2D analysis revealed five proteins (PHB, GANAB, HSPA5, HSP90B1, and HSP90AA1) corresponding to the KEGG pathway known as protein processing in the ER to be highly connected. In the ER, correctly folded proteins are packaged into transport vesicles followed by transferring to the Golgi complex, whereas misfolded proteins are not packaged. Accumulation of such misfolded proteins causes ER stress and activates the unfolded protein response (UPR) signaling pathway for the purpose of preventing further damage [45]. GRP78/BiP is a major ER chaperone protein in the ER stress signaling pathway. When GRP78 is released from the ER, three main branches involving IRE1α, PERK, and ATF6 are activated through interaction with the GRP78 protein. Among them, IRE1α is the only ER stress sensor in yeast and the most conserved branch for the UPR signaling pathway in animals [46]. When IRE1α is activated by interacting with GRP78, IRE1α is autophosphorylated, which generates XBP1s, the spliced form from unspliced XBP1. A transcription factor, XBP1s translocates to the nucleus, where it upregulates the expression of target genes that maintain homeostasis and promote cell survival [45,47,48]. The ER stress signaling pathway is closely connected to autophagy, and the IRE1α and XBP1-mediated ER stress signaling pathway induces autophagy by converting LC3B-I to LC3B-II and enhancing Beclin-1 expression [49]. OXY increased the expression levels of ER stress signaling pathway-related genes and the IRE1α protein. Unlike a MEK1/2 inhibitor, the ER stress inhibitor 4-PBA suppressed the effect of OXY in increasing protein expression of both Atg5 and LC3B. This finding indicates that OXY promotes autophagy via ER stress signaling, resulting in increased Atg5 and LC3B expression.

Mucin is known as the major component in the intestinal mucus layer, and the intestinal mucosal barrier plays a critical role in maintaining intestinal homeostasis and preventing external damage. Autophagy promotes expression of MUC2 [19,50,51], and OXY induces MUC2 expression in intestinal goblet cells [17]. In this study, we found that OXY activates autophagy and that OXY-induced MUC2 expression is decreased by an ER inhibitor. The results suggest that OXY activates autophagy, which might result in stimulation of MUC2 expression.

## 5. Conclusions

Autophagy is known to promote the production of mucins in goblet cells. OXY induces autophagy via ER stress signaling pathways, and OXY-induced autophagy increases MUC2 expression in human LS 174T goblet cells. These findings suggest that OXY may be used for the treatment of intestinal disorders caused by disruption of the intestinal mucus layer through regulation of the autophagy process in the intestinal epithelium.

## Figures and Tables

**Table 1:** Primers used for qPCR analysis.

Gene	Forward (5′ to 3′)	Reverse (5′ to 3′)
*GAPDH*	GAGTCAACGGATTTGGTCGT	GACAAGCTTCCCGTTCTCAG
*ULK1*	CAGAACTACCAGCGCATTGA	TCCACCCAGAGACATCTTCC
*ULK2*	GTGGGAACGTGAGGAAGAGG	CTGGGAGTTTCAGGGTGGTC
*Atg13*	AAAGTCACCTCCCAGTGTGG	GAAAAGCTCCACAGGACAGC
*Atg101*	GAAGTGTGGACGGTCAAGGT	CACGTTATCCACCTCCGACT
*VPS34*	AAGCAGTGCCTGTAGGAGGA	TGTCGATGAGCTTTGGTGAG
*Beclin-1*	AGGTTGAGAAAGGCGAGACA	AATTGTGAGGACACCCAAGC
*Atg7*	GGCCTAGGTACCTGTGACCA	GAAGCTCCCATGAGCTGAAC
*Atg4*	GGCTTACCAAGGGCTACCTC	ACAGCACTGGAAAGGGACAC
*Atg5*	ACCAGAAACACTTCGCTGCT	GACCTTCAGTGGTCCGGTAA
*Atg16L1*	CACAAGAAACGTGGGGAGTT	ACAAAGCTTAGTGCGCAGGT
*LC3B*	CCACACCCAAAGTCCTCACT	CACTGCTGCTTTCCGTAACA
*Rab7*	TGGATGACAGGCTAGTCACG	CTGGCCTGGATGAGAAACTC
*STX17*	GGGTGAAGCCAGGAATGTTA	ATGCCACACCCAGCTAATTC
*VAMP8*	CATCTCCGCAACAAGACAGA	GACCCTCTTGGCACACATTT
*LAMP2*	ACAACTCACTCCACAGGCAG	TGCAATGCTGAAAACGGAGC
*MEK*	GCTTGGGGCTATTTGTGTGT	TCTCACAAGGCTCCCTCCTA
*ERK1*	TCA GAC TCC AAA GCC CTT GAC	CGT GCT GTC TCC TGG AAG ATG
*ERK2*	TCC AAC AGG CCC ATC TTT CC	CCA GAG CTT TGG AGT CAG CA
*IRE1α*	CGGCCTTTGCAGATAGTCTC	ACGTCCCCAGATTCACTGTC
*XBP1*	GGAGTTAAGACAGCGCTTGG	ACTGGGTCCAAGTTGTCCAG
*PERK*	CTCACAGGCAAAGGAAGGAG	AACAACTCCAAAGCCACCAC
*ATF4*	TCAAACCTCATGGGTTCTCC	GAAGGTCATCTGGCATGGTT
*MUC2*	ACCCGCACTATGTCACCTTC	GGACAGGACACCTTGTCGTT

**Table 2:** Proteins identified by 2D gel electrophoresis and peptide mass fingerprinting (PMF) (MALDI-TOF).

Protein	Protein Identification	Fold Change
EIF5A	eukaryotic translation initiation factor 5A-1 isoform B	9.94
PHB	prohibitin	0.003
AP1B1	AP-1 complex subunit beta-1 isoform b	19.206
GANAB	glucosidase, alpha; neutral AB, isoform CRA_a	0.059
HSPA5	GRP78, endoplasmic reticulum chaperone BiP precursor	3.42
IQGAP1	IQGAP1 protein	3.421
ACTN4	alpha-actinin-4 isoform 1	3.994
PDCD6IP	programmed cell death 6-interacting protein isoform 2	0.096
HSP90AB1	heat shock protein HSP 90-beta isoform c	3.464
HSP90B1	tumor rejection antigen (gp96) 1	3.081
HSP90AA1	HSP90AA1 protein	2.469
AHSA1	activator of 90kDa heat shock protein ATPase homolog 1 isoform1	0.149
AARS	alanyl-tRNA synthetase variant	2.356

## Supplementary Materials

The following are available online at https://www.mdpi.com/2076-3921/9/3/214/s1, Figure S1: Western blot data. The full-length blots of Figure 2A. Figure S2: Western blot data. The full-length blots of Figure 3C. Figure S3: Western blot data. The full-length blots of Figure 6B. Figure S4: Western blot data. The full-length blots of Figure 6D. Figure S5: Western blot data. The full-length blots of Figure 7C. Figure S6: Western blot data. The full-length blots of Figure 8B.

## Abbreviations

OXY	oxyresveratrol
LC3	microtubule-associated protein 1A/1B-light chain 3
PE	phosphatidylethanolamine
ATGs	autophagy-related genes
ULK	unc-51-like kinase
PI3K	phosphatidylinositol 3-kinase
RPMI	Roswell Park Memorial Institute
DMSO	dimethyl sulfoxide
3-MA	3-methyladenine
2 DE	two-dimensional electrophoresis
PMF	peptide mass fingerprinting
IEF	isoelectric focusing
DTT	dithiothreitol
ICC	immunocytochemistry
ER	endoplasmic reticulum
4-PBA	4-phenylbutyric acid
