# Supplementary material for: Oxyresveratrol Induces Autophagy via the ER Stress Signaling Pathway, and Oxyresveratrol-Induced Autophagy Stimulates MUC2 Synthesis in Human Goblet Cells
Source: Antioxidants (Basel). 2020 Mar 5;9(3):214. doi: 10.3390/antiox9030214 (PMC7139292; doi:10.3390/antiox9030214)
Supplement: Supplementary file 1 [file antioxidants-09-00214-s001.pdf]

Supplementary materials

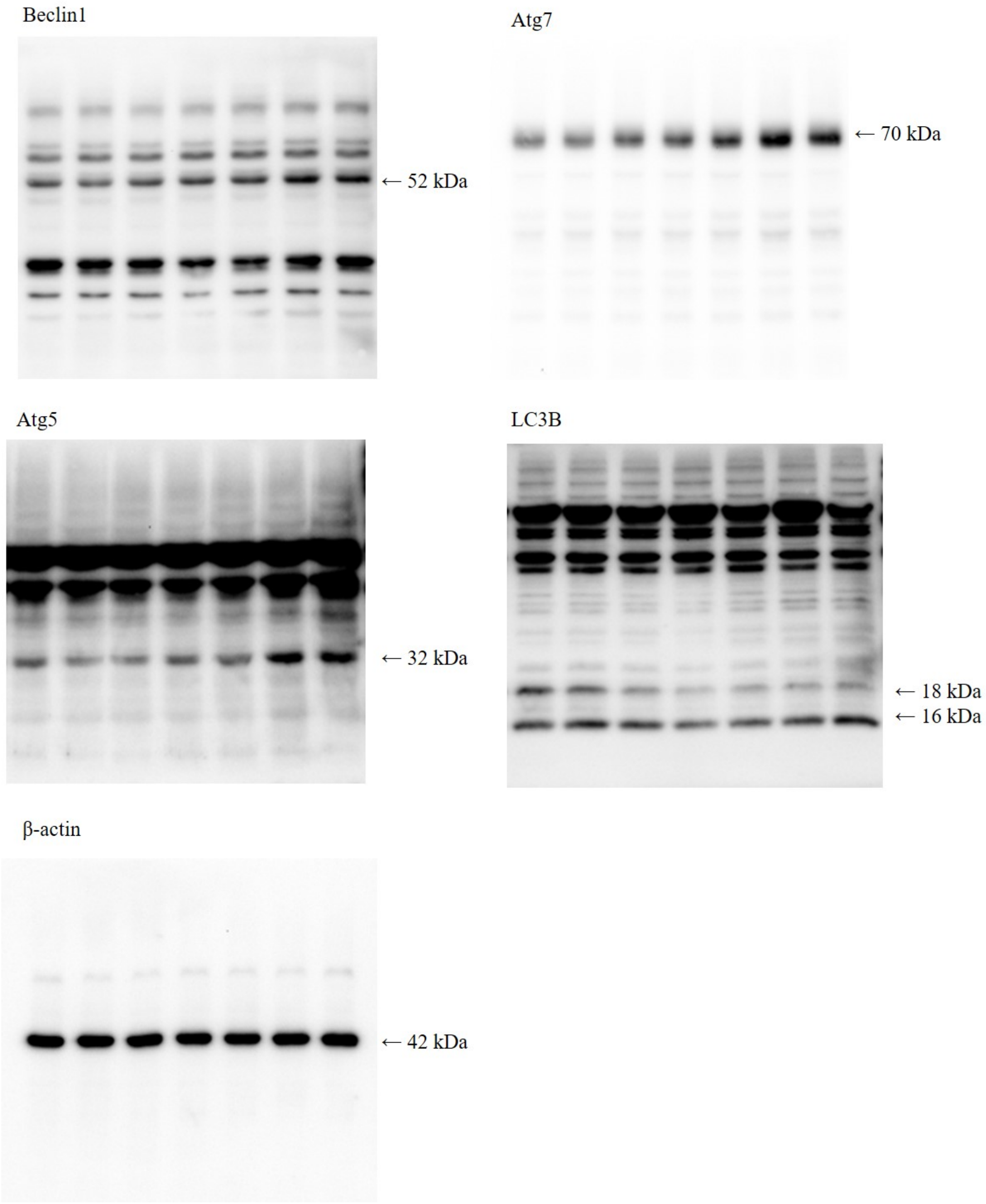

Figure S1. Western blot data. The full-length blots of Figure 2A.

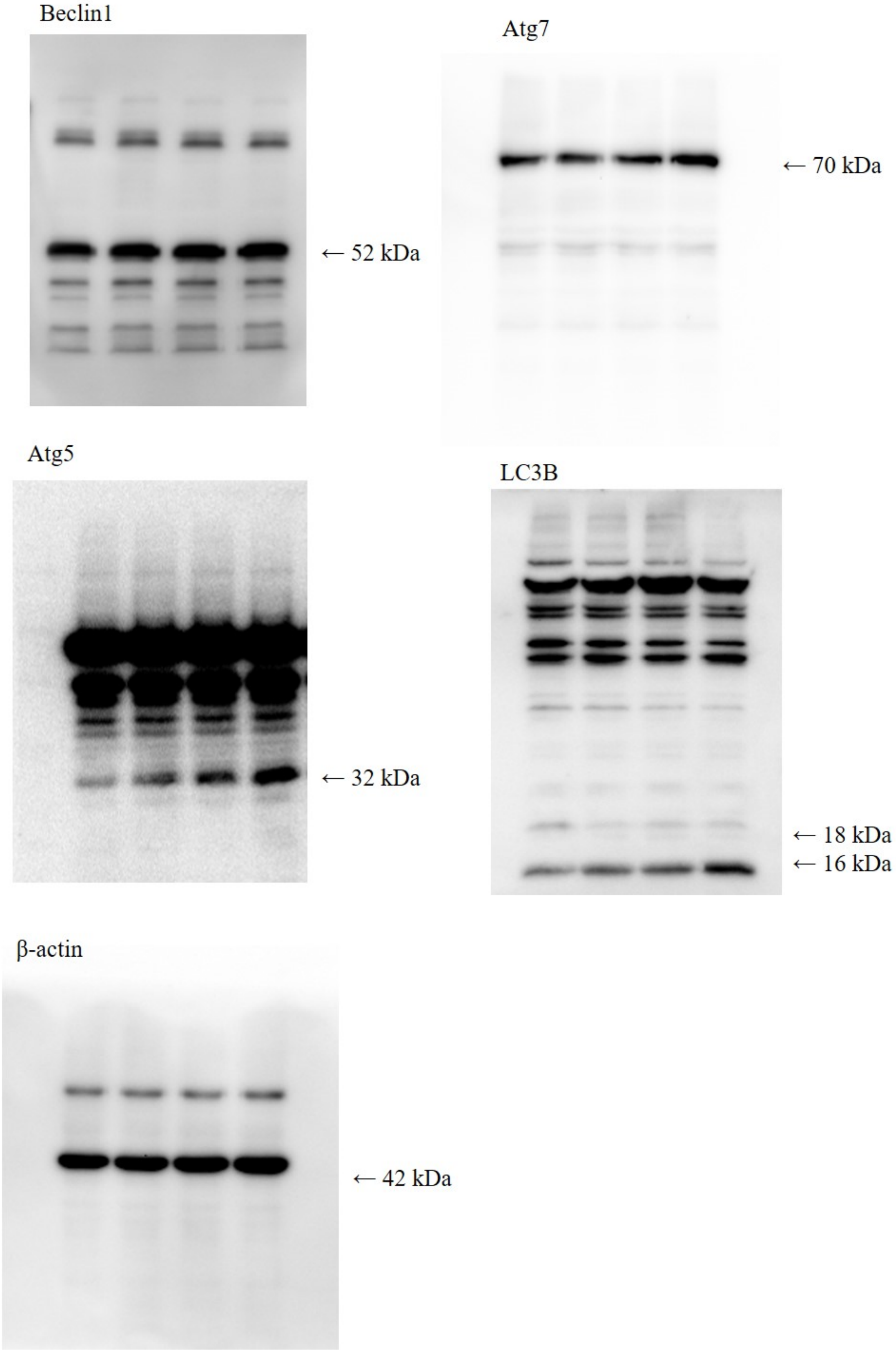

Figure S2. Western blot data. The full-length blots of Figure 3C.

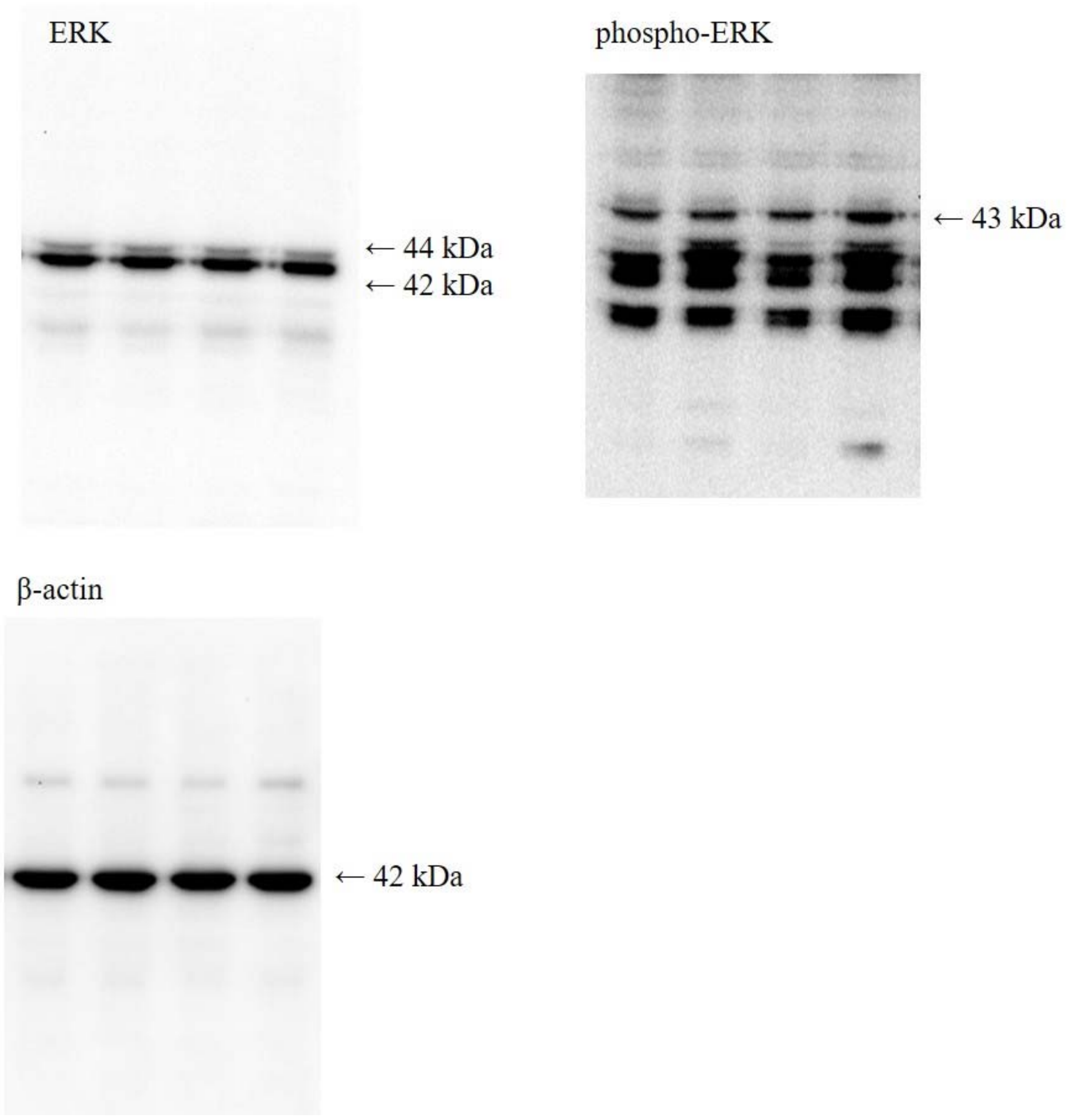

Figure S3. Western blot data. The full-length blots of Figure 6B.

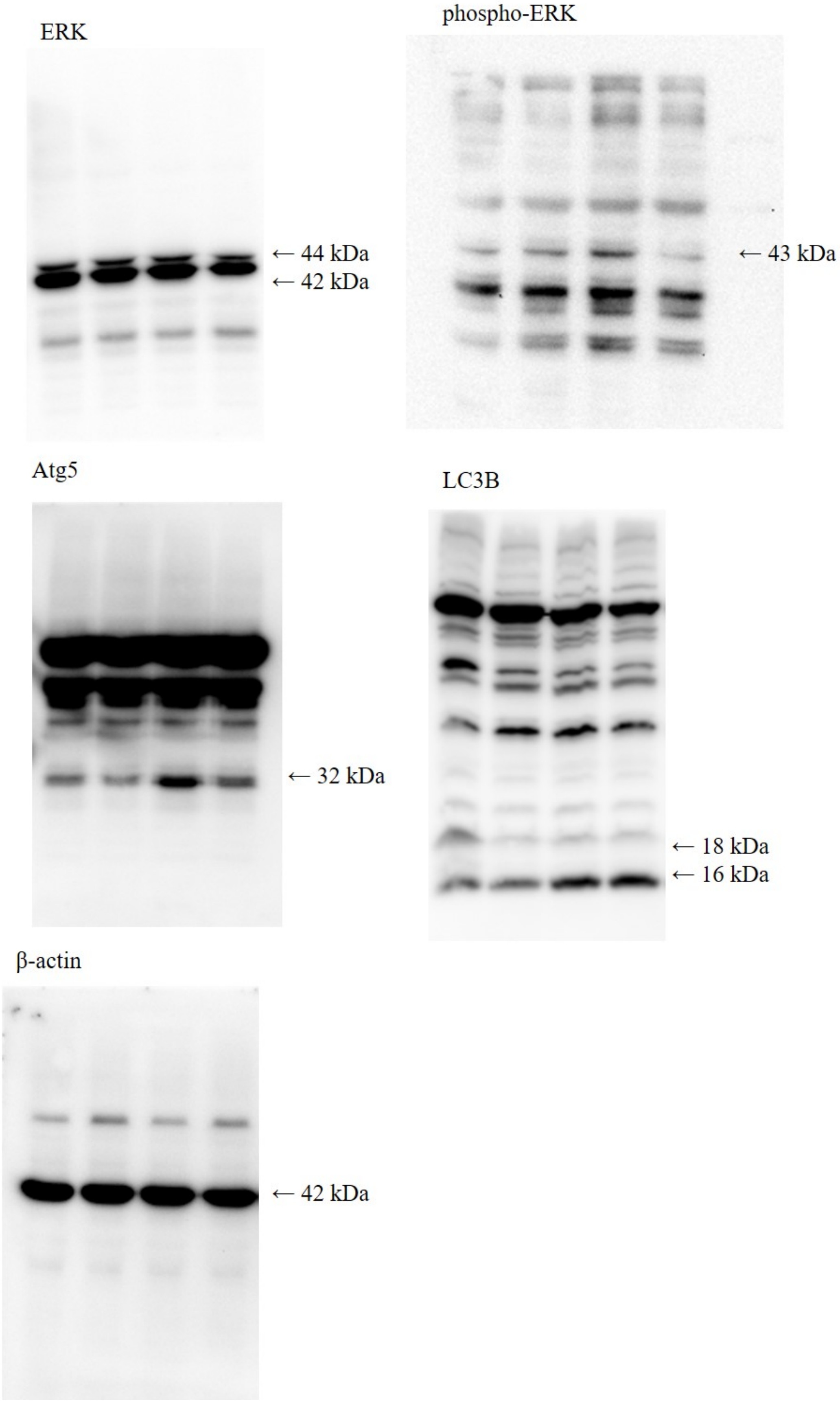

Figure S4. Western blot data. The full-length blots of Figure 6D.

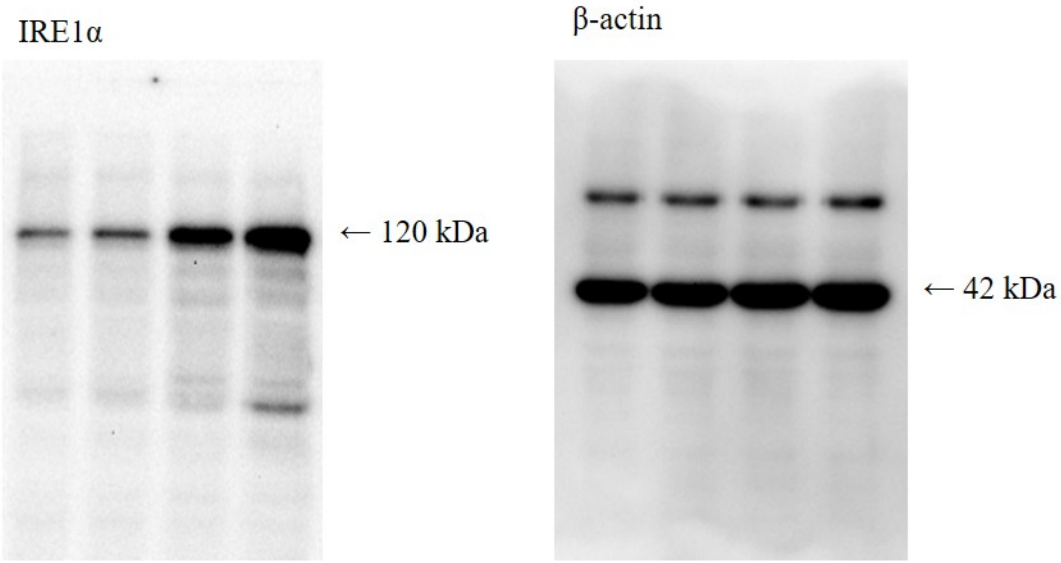

Figure S5. Western blot data. The full-length blots of Figure 7C.

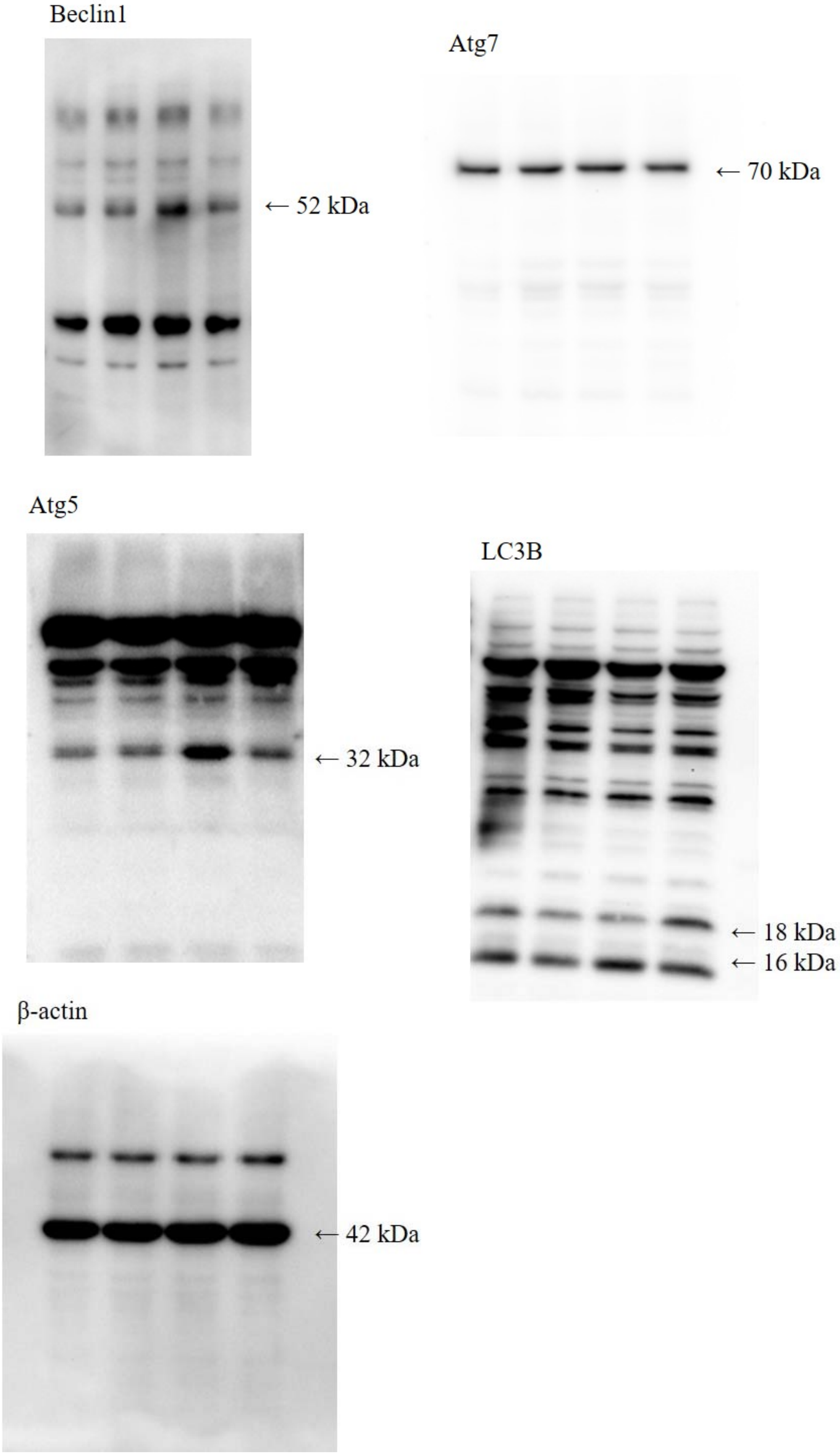

Figure S6. Western blot data. The full-length blots of Figure 8B.
